# High pressure processing of hummus: Enhancing microbial safety and stability, and reducing lipid oxidation

**DOI:** 10.1016/j.heliyon.2025.e42590

**Published:** 2025-02-10

**Authors:** Tareq M. Osaili, Dinesh Kumar Dhanasekaran, Fayeza Hasan, Reyad S. Obaid, Anas A. Al-Nabulsi, Amin N. Olaimat, Leila Cheikh Ismail, Hayder Hasan, Mutamed Ayyash, Gafar Babatunde Bamigbade, John Ortiz, Richard Holley

**Affiliations:** a Department of Clinical Nutrition and Dietetics, College of Health Sciences, University of Sharjah, P. O. Box 27272, Sharjah, United Arab Emirates; b Research Institute of Medical & Health Sciences, University of Sharjah, P. O. Box 27272, Sharjah, United Arab Emirates; c Department of Nutrition and Food Technology, Faculty of Agriculture, Jordan University of Science and Technology, P.O. Box 3030, Irbid, 22110, Jordan; d Department of Clinical Nutrition and Dietetics, Faculty of Applied Medical Sciences, The Hashemite University, Zarqa, Jordan; e Department of Women's and Reproductive Health, University of Oxford, Oxford, OX39DU, UK; f Department of Food, Nutrition and Health, College of Food and Agriculture, United Arab Emirates University (UAEU), United Arab Emirates; g Smartfood Solutions FZCO, Dubai Silicon Oasis, P.O. Box 341147, Dubai, United Arab Emirates; h Department of Food Science and Human Nutrition, University of Manitoba, Winnipeg, Manitoba, R3T 2N2, Canada

**Keywords:** Chickpeas, Nonthermal processing, Shelf life, *Salmonella*, *E. coli* O157:H7, *L. monocytogenes*

## Abstract

Hummus provides an ideal environment for microbial growth. The objectives of this study were to evaluate the effect of high-pressure processing (HPP) on i) microbial safety/quality, ii) physical/chemical properties, and iii) sensory characteristics of hummus. Uninoculated and hummus inoculated with *Salmonella* spp., *Escherichia coli* O157:H7, and *Listeria monocytogenes* were subjected to HPP at 350 MPa for 1–5 min. After treatment, the D-value of the pathogens was calculated and uninoculated samples were stored for up to 28 d at 4 and 10 °C and total microbial counts (TMC) were enumerated. Thiobarbituric acid reactive substances (TBARS), colour, textural and rheological properties and sensory characteristics of hummus were also analysed. The D_10_-value for *Salmonella* spp., *E. coli* O157:H7 spp. and *L. monocytogenes* were 2.10 ± 0.13, 1.48 ± 0.08, and 3.77 ± 0.36 min, respectively. As compared to the control, HPP for 1, 2, 3, 4, and 5 min instantly decreased TMC on average by 0.7, 1.2, 1.6, 1.4 and 1.8 log cfu/g, respectively. The shelf life of hummus in this study after an HPP treatment of 350 MPa for 2–5 min was 28 d at 4^o^C and one week at 10 °C, while it was 14 d and 7 d in the control samples, respectively. HPP decreased TBARS but did not significantly change hummus lightness, greenness, and yellowness. HPP enhanced the gel strength and viscoelastic properties of hummus without compromising its sensory qualities. Thereby, HPP at 350 MPa for 1–5 min can be effective and adopted by producers.

## 1 Introduction

Hummus is a popular Middle Eastern dip prepared by boiling and mashing chickpeas which are mixed with tahini (sesame paste), lemon juice or citric acid, garlic, in addition to salt and served with olive oil [1]. Hummus is a rich source of nutrients that includes complex carbohydrates (14.3g/100g), plant-based proteins (7.9g/100g), and healthy fats (9.6g/100g) [2]. It is also low in sugar, saturated fat, and calories. Hummus is served as a ready-to-eat product.

In the recent past, several outbreaks of foodborne illness associated with hummus have been reported. A *Salmonella*-associated outbreak caused by hummus (tahini) was reported by the CDC which generated 23 illnesses and one death. Another outbreak due to *Salmonella* contamination of hummus involved 802 cases [3]. Similar outbreaks have been reported in Australia and New Zealand [4,5]. Pathogens like *Escherichia coli* and *Listeria monocytogenes* have also been identified in hummus [6,7].

The high carbohydrate content and water activity (0.95–0.98) of hummus provides an ideal environment for microbial proliferation [8,9], and since chickpeas are the only ingredient which receives a heat treatment (boiling), it is not surprising that its shelf life is limited to 24–72 h at refrigeration temperatures [10]. Cross-contamination of hummus may occur after boiling, from other ingredients, utensils, handlers, or the environment [10]. The presence of microorganisms such as lactic acid bacteria, *Enterobacteriaceae*, *Pseudomonas*, and yeast and molds can result in premature spoilage [11].This is why hummus is rarely produced in large quantities at the restaurant/home level. There is limited capacity to produce fresh hummus for export purposes. Nonetheless, due to its healthy nature, hummus consumption has increased globally with overall value of trade reaching $2 billion (US) in 2017 [12].

Over the past few years, high-pressure processing (HPP), also known as cold pasteurization, has gained popularity. This nonthermal processing technique subjects food items to elevated pressures, with water as the transmission medium. Commercially, it can involve pressures of up to 600 MPa, while at the laboratory scale where smaller HPP vessels are used, pressures of up to 1000 MPa can be safely reached. Previous studies have indicated that upon appropriate processing conditions, HPP can improve microbial quality without affecting the organoleptic properties of some foods [13,14].The same, however, cannot be said for lipids, because kinetic reactions of these compounds change under high pressure. HPP may also cause protein degradation which can release prosthetic groups or free radicals [15]. HPP also affects bond formation, charge separation and concentration, and steric crowding which may all affect lipid oxidation. Furthermore, textural properties, especially hardness, are influenced by particle size, which is determined by chickpea proteins which interact with oil to create the weak gel network structure responsible for texture development. Additionally, the breakdown of the large molecular weight particles into smaller aggregates has a significant effect on textural properties [16]. A review conducted previously indicated that HPP impacts factors like flocculation rate, crystallization, and coalescence in food emulsions [17].

It is with this background in mind that the current work to improve hummus shelf life, stability, quality, and safety by HPP was undertaken. The objectives of this study were to evaluate the effect of HPP on i) the microbial safety/quality ii) physical/chemical properties and iii) sensory characteristics of hummus.

## 2 Materials and methods

### 2.1 Preparation of the hummus

Fresh hummus was prepared before each trial using sorted chickpeas soaked overnight in potable water for 8 h. Soaked chickpeas were boiled with sodium bicarbonate (0.83 %), cooled with water and then spread on a stainless steel tray for 90 min at 4 °C. The proportion of ingredients used in the recipe was as follows: chickpeas (50 %) 1050 g, tahini (16.4 %) 345 g, NaCl (0.6 %) 12.6 g, citric acid (0.7 %) 15 g and water (32.3 %) 677.4 g. All ingredients were crushed with a sterile blade into a smooth paste, yielding a total weight/batch of 2.1 kg [18]. The initial pH and water activity of hummus samples were measured using a pH meter (Cyberscan 500; Eutech Instruments, Singapore) and a water activity meter (Hygrolab, Rotronic Instrument Corp., Hauppauge, NY, USA).

### 2.2 Bacterial strains and culture preparation

Two food isolates of *Salmonella* (*S*. Copenhagen PT 99 and *S*. Typhimurium 02–8423), *E. coli* O157:H7 (strains 1934 and 161‐84) and *L. monocytogenes* (L. M. 7644 and L.M. GLM 5) were used in the current study. The cultures were activated as in a previously published protocol [18]. A cocktail of each genus was formed by mixing 1 ml from the individual strains or species to reach a level of 7 log cfu/ml.

### 2.3 Pathogen inoculation and hummus treatment

A volume of 1 ml of each pathogen cocktail was individually mixed with 100 g of the prepared hummus. Samples weighing 25 g were placed into sterile plastic sous-vide vacuum pouches (20 cm × 15 cm) and vacuum packaged (Henkelman B.V., 's-Hertogenbosch, The Netherlands). To identify an appropriate pressure for the experiment, several experiments were conducted using various pressure levels to assess the reduction rates of microorganisms. The samples were treated at a hyperbaric pressure of 350–450 MPa operated at 10 °C (Hiperbaric 55, Burgos, Spain) at hold times of 1, 2, 3, 4, or 5 min. All analyses were conducted in triplicate. Three 2.1 kg batches of hummus were prepared for each test.

Uninoculated hummus samples were prepared in the same way for background microbiota enumeration (total plate counts, TMC). TMC was used as an index of microbial quality and shelf-life determination of food [19]. After the treatment, uninoculated hummus samples were stored at refrigerated (4 °C) or abusive (10 °C) temperatures for 1, 7, 14, 21 and 28 d. Non-treated hummus samples served as the control in the experiments.

### 2.4 Microbial enumeration

Control and HPP-treated samples were aseptically added to 75 ml sterile peptone water (Himedia, Maharashtra, India) and mixed (Interscience, Paris, France) for 2 min. Appropriate serial dilutions followed by enumeration were performed according to methods used in previous studies [20]. Total microbial counts (TMC), *Salmonella* spp., *E. coli* O157:H7 and *L. monocytogenes* were enumerated on Plate Count Agar at 30 °C for 3 d (Himedia), Xylose Lysine Deoxycholate Agar at 37 °C for 36 h (Himedia), Sorbitol MacConkey Agar with Cefixime-Tellurite Supplement at 37 °C for 48 h (Himedia) and Listeria Selective Agar with Modified Listeria Selective Supplement at 37 °C for 48 h (Himedia), respectively. Total microbial count was measured in the uninoculated samples stored at 4 °C and 10 °C for 0, 1, 7, 14, 21 and 28 d.

### 2.5 D-value calculation

The D-value for *Salmonella* spp., *E. coli* O157:H7, and *L. monocytogenes* in HPP-treated hummus was calculated using a survival curve [21].

$$D-value=\frac{t_{2}-t_{1}}{\log_{A}-\log_{B}}$$

Where A and B represent the surviving numbers after HPP treatment for times t_1_ and t_2_, respectively.

### 2.6 Thiobarbituric acid reactive substances assay (TBARS)

The quantity of reactive substances in the hummus samples produced by fatty acid oxidation was measured by monitoring TBARS [22]. Briefly, a thiobarbituric acid (TBA) solution was prepared from a mixture of 15 % of trichloroacetic acid (TCA), 0.375 % TBA, 2 % 0.25 N HCl and deionized water to make up to the desired volume. The TBA solution and hummus sample were mixed in a 5:1 ratio and vortex-mixed. The mixture was heated in boiling water for 10 min to form a pink colour. It was cooled at room temperature, centrifuged at 10,000 rpm (2240 *g*) for 15 min, and the absorbance of the supernatant was read at 532 nm using a UV spectrophotometer (Shimadzu UV-1800, Kyoto, Japan). Water was used in place of the sample to serve as a control and tests were performed in triplicate. The absorbances were compared with a standard curve prepared from 1,1,3,3-tetramethoxypropane (MAD) from concentrations of 0–10 ppm. The TBARS results were expressed as mg of MAD equivalents/kg of sample.

### 2.7 Colour measurement

The colour of the hummus samples was measured at room temperature using a Hunterlab ColourFlex EZ Colourimeter (HunterLab, Reston, VA20190, USA) equipped with a 5 cm diameter aperture. All sample colours were measured in triplicate and results were interpreted with the CIELAB colour space reference system using *L∗L∗* (black and white representing 0 and 100, respectively), *A∗* (red (>0) to green (<0) colour range) and *B∗* (yellow (>0) to blue (<0) colour range) [23].

### 2.8 Texture profile analysis (TPA)

The TPA of the hummus samples was evaluated with measurements of hardness, adhesiveness, and stringiness using a texture analyser CT3 (Brookfield Ametek, Harlow, UK) equipped with a 25 mm cylindrical probe [16]. Samples weighing 100 g each were treated in a cylindrical container with a load cell of 4.5 kg using a trigger force of 6.8 g at a 0.5 mm/s test speed to monitor deformation over a 10.0 mm target distance. The selected textural parameters were assessed in triplicate and the TPA was obtained using TexturePro software (Brookfield Instruments, Harlow, UK).

### 2.9 Rheological properties

Hummus samples weighing 3 g were subjected to three tests using a rheometer (Discovery Hybrid HR-2, TA Instruments, New Castle, DE, USA) equipped with a parallel geometry stainless steel plate having a diameter of 40 mm set at a gap of 500 μm, and a plate-controlled temperature of 25 ± 0.1 °C. All data were analysed using TRIOS 5.2 software (TA Instruments).

#### 2.9.1 Strain and frequency sweep tests

The linear viscoelastic region of the hummus samples was evaluated using the strain sweep test in the strain range of 0.01–10 % at a constant frequency of 1.0 Hz. The samples exhibited linearity up to 0.3 % which was then chosen for subsequent tests. Similarly, the frequency sweep test was used to determine the viscoelastic behaviour of the hummus samples at a frequency range of 0.1 and 50 Hz and a constant strain of 0.3 % within the linear viscoelastic region.

#### 2.9.2 Time-dependent behaviour

The thixotropic behaviour of the hummus samples was measured with low and high shear conditions to determine structural deformation and recovery [2]. The storage (G′) and loss (G″) moduli were measured using an oscillation-time test at a constant frequency of 1.0 Hz over three time segments: firstly (200 s, 0.3 % strain), secondly (60 s, 50 % strain), and thirdly (400 s, 0.3 % strain).

### 2.10 Sensory analysis

The sensory evaluation trials involved 40 untrained panellists who were selected based on the snowball sampling technique [24]. They were aged between 20 and 45 years and were familiar with hummus consumption. The panellists were asked to rate the colour, texture, taste, flavour, and overall acceptability of uninoculated hummus samples treated with HPP at 350 MPa for 1, 2, 3, 4 or 5 min using a 9-point hedonic scale (9 = like extremely, to 1 = dislike extremely) [25].

### 2.11 Statistical analysis

The effect of treatment, storage time, and their interactions on microbial populations was quantified using two‐way analysis of variance (ANOVA) and post hoc analysis by Tukey's HSD (IBM SPSS Statistics software, version 26, Chicago, IL, USA). The effect of storage temperatures on the microbial populations was quantified using two-tailed, unpaired student t-tests with GraphPad Prism Version 7.0 (GraphPad Software, Inc., Boston, MA, USA). Results of sensory evaluation, colour, texture and TBARS of hummus samples were analysed for One-way ANOVA and post-hoc analysis by Tukey HSD. A significance value of *p* < 0.05 was established for the statistical analysis.

## 3 Results and discussion

### 3.1 Effect of HPP on microbial quality and shelf life

In the current study, only a pressure of 350 MPA was applied. Higher pressure levels (400–450 MPa) were not selected because the tested pathogenic microorganisms exhibited an exceptionally high reduction rate at these pressures, resulting in their rapid inactivation within seconds. As a results, it was not feasible to gather sufficient data to analyze reduction behavoir or calculate the D-values of the tested bacteria.

The initial water activity and pH of hummus were 0.98 ± 0.00 and 4.4 ± 0.0. The initial TMC counts in the control samples at 4 and 10 °C were 4.68 ± 0.31 and 4.39 ± 0.34. HPP for 1–5 min decreased the TMC populations in hummus samples after treatment (day 0) between 0.5 and 1.9 log cfu/g compared to the control samples (Table 1). Similarly, HPP of aronia berry juice at 400 MPa for 5 min decreased TMC by 2 log cfu/g [26]. In other work, HPP of mango pulp at 400 MPa for about a minute decreased total aerobic mesophiles by about 4 log cfu/g [27].

**Table 1:** Changes in total microbial counts (log_10_ ± SD CFU/g) in hummus samples after 350 MPa HPP treatment and storage at 4 °C or 10 °C for 28 d.

Temp (°C)	Days	0 min	1 min	2 min	3 min	4 min	5 min	*P - value*
4	0	4.7^cde^ ± 0.0	3.8^defghij^±0.7	3.3^efghijk^±0.6	3.1^ghijkl^±0.6	3.5^efghijk^±0.6	3.0^hijkl^±0.6	0.001
4	7	5.0^cd^ ± 0.8	4.4^defg^±0.9	3.2^fghijkl^±0.8	3.1^fghijkl^±0.6	3.0^hijkl^±0.6	2.6 ^jkl^ ± 0.1	
4	14	6.7^ab^ ± 0.9	2.9^ijkl^±0.5	4.5^cdef^±0.2	3.7^defghij^±0.7	4.0^defghi^±0.6	4.0^defghi^±0.7	
4	21	7.4^a^±0.3	6.7^ab^ ± 0.9	4.3^defgh^±0.2	5.8^bc^±0.1	3.2^fghijkl^±0.6	3.4^efghijk^±0.5	
4	28	6.6^ab^ ± 0.7	6.5^b^ ± 0.4	2.5^jkl^ ± 0.2	2.7^ijkl^±0.3	2.4^kl^ ± 0.3	1.9^l^ ± 0.3	
10	0	4.4^c^±0.0	3.9^cd^ ± 0.4	3.3^cde^ ± 0.3	2.9^cde^ ± 0.0	2.9^cde^ ± 0.0	2.5^de^ ± 0.8	0.001
10	7	6.9^b^ ± 0.7	4.1^c^±0.1	3.9^cd^ ± 1.0	2.5^de^ ± 0.3	2.3^e^±0.2	2.4^de^ ± 0.0	
10	14	8.2^ab^ ± 0.3	7.8^ab^ ± 0.4	7.6^ab^ ± 0.8	8.1^ab^ ± 0.2	7.6^ab^ ± 0.7	7.7^ab^ ± 0.7	
10	21	8.7^a^±0.6	8.7^a^±0.8	8.5 ± 1.0	8.7^a^±0.9	8.5^a^±0.9	8.2^ab^ ± 0.3	
10	28	7.8^ab^ ± 0.4	7.8^ab^ ± 0.4	7.4^ab^ ± 0.6	7.8^ab^ ± 0.4	7.5^ab^ ± 0.6	7.3^ab^ ± 0.8	
4 °C vs 10 °C	0	NS	0.214	0.277	0.405	0.063	0.732	
	7	0.001	0.168	0.188	0.209	0.174	0.010	
	14	0.000	0.000	0.000	0.000	0.001	0.000	
	21	0.000	0.001	0.000	0.000	0.000	0.000	
	28	0.017	0.001	0.000	0.000	0.000	0.000	

^a,b,c,d,e,f,g,h,I,j,k,l,^ Different letters in each packaging condition indicate significant differences (*p* < 0.05) among the means.

∗Different letters in each column indicate a significant difference (*p* < 0.05) between the means at the same day of storage at 4 °C or 10 °C.

It should be noted in the present study that the faster increase in TMC numbers in HPP-treated samples during storage at 10 °C than at 4 °C was expected [28]because it was anticipated that 350 MPa treatment for up to 5 min would injure, rather than kill contaminating bacteria [29]. Abusive storage at 10 °C would have provided better conditions for proliferation than 4 °C. The subsequent increase in TMC populations followed by a decline may be explained by competition amongst the bacteria for limited resources [30].

After 28 d storage at 4 and 10 °C, the population in control samples increased by 1.9 and 3.4 log cfu/g, respectively (Table 1) and was beyond the usual accepted shelf-life limit of 6 log cfu/g [31]. However, in samples that already received the HPP treatment and were stored at 4 °C for 28 d, decreases of 0.8 (2 min), 0.4 (3 min), 1.2 (4 min) and 1.1 (5 min) log cfu/g were observed (as a result of refrigeration). Considering an accepted shelf-life limit of 6 log cfu/g, the shelf life of hummus in this study after an HPP treatment of 350 MPa for 2–5 min was 28 d at 4^o^C. This was only less than that found in refrigerated humus preserved with 0.05 % potassium sorbate, 0.10 % sodium benzoate or 0.05 % sodium metabisulfite. The shelf life of these products was 35 d, 35 d, or 63 d, respectively, but upon storage at 10 °C, the shelf life was only 13 d, 7 d, or 11 d, respectively [10]. In the present study at 10 °C the TMC in HPP-treated samples after 28 d storage increased by 3.9 (1 min), 4.1 (2 min), 4.8 (3 min), 4.6 (4 min), 4.8 (5 min) log cfu/g. Product shelf life was only one week at this temperature.

### 3.2 Effect of HPP on pathogen elimination

**Fig. 1:**
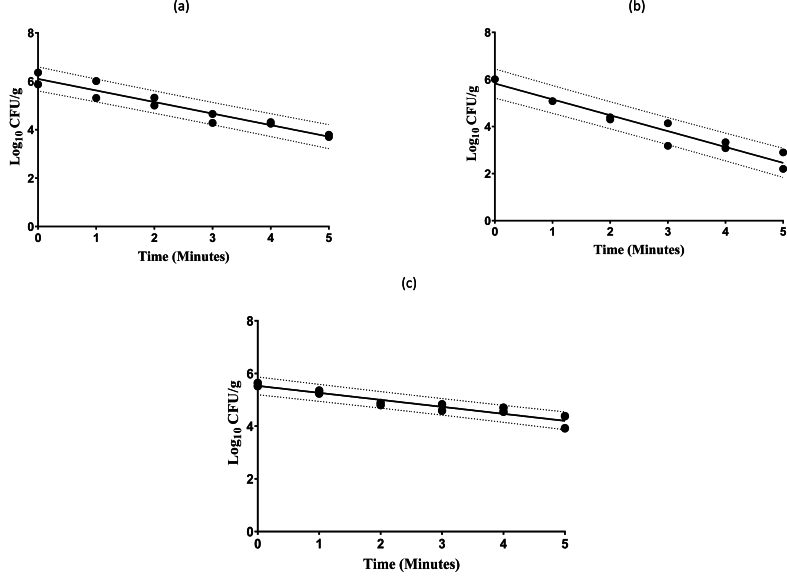
Survival curve of **(a) *Salmonella* spp.** (R^2^ = 0.9305, correlation coefficient = −0.477) **(b) *E. coli* O157:H7** (R^2^ = 0.945, correlation coefficient = −0.675) and (**c) *L. monocytogenes*** (R^2^ = 0.901, correlation coefficient = −0.265) with confidence prediction bands represented by the paired dotted lines. Hummus samples were treated at 350 MPa with HPP.

The D-values for *Salmonella* spp., *E. coli* O157:H7 spp. and *L. monocytogenes* were 2.10 ± 0.13, 1.48 ± 0.08, and 3.77 ± 0.36 min at 350 MPa HPP for hold times of 1–5 min, respectively [Fig. 1A - CA - C].

In a previous study, a treatment of 400–450 MPa decreased *Salmonella* in salsa by approximately 6 log cfu/g [32]. It was also observed that the pH and water activity, besides the type of acidulant (vinegar or lemon juice), affected the extent of inactivation in the salsa. The difference in water content may explain the lower inactivation rates in hummus.

Meanwhile, in this study, HPP treatment at 350 MPa for 5 min decreased *E. coli* O157:H7 by 3 log cfu/g. In comparison, treatment at 300 MPa for 5 min decreased *E. coli* by 4 and 1 log cfu/ml in kiwi fruit juice and pineapple juice, respectively [33]. This is an interesting observation as the microbial reduction in juices was expected to be significantly higher than that in hummus due to their lower pH and higher water activity.

In a study conducted previously on hummus, also at 350 MPa, the D-value for *L. monocytogenes* was observed to be 1.8 min [34]. The difference in results may have been due the use of different strains of *L. monocytogenes* [35] and differences in product composition.

As for L. *monocytogenes* spp., in the current study HPP at 350 MPa for 5 min decreased the pathogen by 1 log cfu/g. In a previous study, HPP of hummus, guacamole, and baba ghanoush at 350 MPa for 4 min decreased *L. monocytogenes* by 1, 1, and 5 log cfu/g, respectively [34]. However, treatment using the same parameters did not decrease *L. monocytogenes* in tahini and pesto. This clearly indicates that the type of food matrix can influence the success of pathogen inactivation. A study previously conducted on *L. monocytogenes* indicated that a lower water activity helped in stabilizing structural proteins. This made the proteins resistant to any form of denaturation because of pressure and indirectly aided in cell survival [36]. Preliminary research in meat products indicates that in comparison to fat content, water activity plays a bigger role in the inactivation of pathogens [37].

### 3.3 Effect of HPP on hummus TBARS

Lipid oxidation may affect the organoleptic properties of foods that contain vulnerable lipids. Lipid peroxidation (primarily malondialdehyde) can be measured using TBARS. In this study, HPP of hummus samples significantly (*p* < 0.001) decreased lipid peroxidation; however, the duration of the treatment did not have a significant impact (Table 2). HPP can decrease lipid oxidation in hummus primarily by inactivating oxidative enzymes such as lipoxygenase and reducing the activity of spoilage microorganisms that produce these enzymes, thereby slowing the oxidation process [38]. This contrasts with a study conducted on pesto sauce where HPP did not significantly affect the TBARS value [39]. Results might be explained by differences in lipid composition as well as differences in the matrices of the two foods examined.

**Table 2:** TBARS content (MDA eq/kg sample) in hummus samples treated with 350 MPa HPP for different intervals (min).

	Time (min)						
	0	1	2	3	4	5	*P* -Value
**TBAR** content (MDA eq/kg sample)	13.18 ± 1.59^a^	5.65 ± 0.48^b^	5.22 ± 0.15^b^	5.21 ± 0.15^b^	4.96 ± 0.15^b^	4.87 ± 025^b^	*p* < 0.001

**∗**Different letters in each treatment indicate significant differences (*p* < 0.05) among the means.

### 3.4 Effect of HPP on hummus colour

The lightness (*L∗*) of the hummus samples did not change significantly (P = 0.723) upon HPP treatment as compared to the control (*p* > 0.05) (Table 3). This result indicating that the brightness of the hummus remained stable. However, the *A∗* (red-green axis) parameter significantly (*p* < 0.05) increased to 1.58 after 1 min of HPP treatment compared to the control and it increased further after 2 min of HPP treatment to 1.82. This means the hummus appeared redder post-HPP treatment compared to the control, after which there was no significant difference regardless of treatment time. There was also a significant increase in the *B∗* (yellow-blue axis) parameter after 1 min of treatment compared to the control which made it appear more yellow. However, a general range of around 22.0 was observed regardless of treatment time. The observed colour changes in hummus following HPP treatment might be attributed to alterations in food chemistry under high pressure, such as pigment oxidation or other molecular changes [40]. The results align with another study conducted on hummus made with broccoli and chickpeas where no significant difference in *L∗* and *B∗* parameters were observed after HPP treatment at 550 MPa for 10 min [41].

**Table 3:** Colour parameter results among hummus samples treated at 350 MPa by HPP.

Parameter	Time (min)						
	0	1	2	3	4	5	*p* – value^a^
***L∗***	79.95 ± 0.21	80.03 ± 0.15	80.08 ± 0.13	80.07 ± 0.21	80.04 ± 0.09	79.89 ± 0.19	0.723
***A∗***	1.44 ± 0.03^c^	1.58 ± 0.04^b^	1.82 ± 0.01^a^	1.84 ± 0.03^a^	1.85 ± 0.05^a^	1.89 ± 0.03^a^	<0.001
***B∗***	21.68 ± 0.10^d^	22.00 ± 0.07^c^	22.01 ± 0.04^bc^	22.10 ± 0.03^bc^	22.42 ± 0.11^a^	22.20 ± 0.03^b^	<0.001

a Significant difference (*p* < 0.05).

### 3.5 Effect of HPP on hummus texture

When hummus samples were subjected to a 4 min hold during HPP treatment, their hardness became significantly greater and reached a maximum value of 43.8 (Table 4). This suggests that the HPP hold time had a significant effect on the structural matrix of hummus samples, causing the formation of stronger protein networks which yielded a gel with improved strength and density. This structural modification can be attributed mainly to HPP treatment penetrating the thin oil-coating barrier of hummus and/or denaturation of the emulsion formed [16]. Additionally, various HPP treatments ranging from low to high have been reported to significantly influence the texture profile analysis of cooked kabuli chickpea samples [42]. It is worth mentioning that there was a significant decline in the hardness of the hummus samples when subjected to 5 min of HPP treatment. This may have been due to pressurization causing an unfolding of more proteins resulting in an increased interaction with water and other components since there was evidence of complete starch gelatinization during cooking. Several studies have correlated increased pressure with denaturation and unfolding of plant proteins which consequently distort the typical protein structure [16,43,44]. There was a significant reduction in the firmness and hardness of chickpea samples treated with 600 MPa while there was no change in those treated with 200 and 400 MPa [42]. This phenomenon can be explained by tissue collapse, internal moisture redistribution and attenuation of hydrophilic bonds of the native protein. In contrast, a significant increase infirmness and hardness was observed in uncooked chickpeas which might have been due to protein aggregation in the samples [45].

**Table 4:** Effect of 350 MPa HPP treatment on hummus texture.

Time (min)						
Parameter	0	1	2	3	4	5
**Hardness**	21.6 ± 3.6^d^	30.5 ± 1.6^c^	33.0 ± 2.8^b^	39.9 ± 1.4^ab^	43.8 ± 5.2^a^	34.3 ± 2.3^b^
**Adhesiveness (mJ)**	1.1 ± 0.2^c^	1.4 ± 0.2^b^	1.3 ± 0.2^b^	1.6 ± 0.1^a^	1.6 ± 0.1^a^	1.4 ± 0.0^b^
**Stringiness (mm)**	11.4 ± 1.7^a^	11.2 ± 0.8^a^	10.4 ± 0.6^ab^	10.2 ± 0.3^b^	9.5 ± 0.8^c^	10.3 ± 0.8^ab^

^a,b,c,d^ Different letters in each packaging condition indicate significant differences (*p* < 0.05) among the means.

It was noted that the adhesive profile showed both an increasing and decreasing trend with an increase in hold time. Specifically, the adhesive profile significantly (*p* < 0.05) increased from 1.1 mJ in the control to 1.3 mJ in 2 min, then 1.6 mJ in 3 min and 4 min before decreasing to 1.4 mJ at 5 min which was comparable to the 2 min sample. Stringiness is an important textural parameter that reveals the sample's ability and rate to recover following deformation. It is directly proportional to the work done to break the internal force joining the particle network [46]. Hence, the higher the value, the lower the disruption of the internal force of the hummus samples. The stringiness of the HPP-treated hummus samples showed a trend like the adhesive parameter which ranged from 9.5 mm to 11.4 mm for 4 min and control, respectively. There were no significant differences (*p* > 0.05) between the stringiness parameter of control and 1 min as well as 2 min and 5 min samples. There was a significant difference (*p* < 0.05) in the stringiness values at 3 min (10.2 mm) and 4 min (9.5 mm) which may have been related to the effect of the HPP hold time. Furthermore, the results also showed that the stringiness values were inversely proportional to the hardness parameters, hence, the higher the hardness, the lower were the stringy properties of the hummus [16,47].

### 3.6 Effect of HPP on hummus rheological properties

**Fig. 2:**
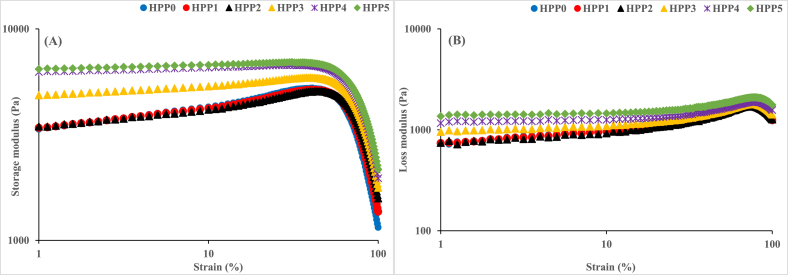
Linear test of hummus samples treated with 350 MPa HPP. **HPP 0:** no treatment (control), **HPP 1:** treatment for 1 min, **HPP 2:** treatment for 2 min, **HPP 3:** treatment for 3 min, **HPP 4:** treatment for 4 min, **HPP 5:** treatment for 5 min.

Fig. 2A and B shows the results of the strain sweep test conducted to determine the linear viscoelastic region (LVR) of the HPP-treated hummus samples. The results show that the system maintained a linear trend for both storage (G′) (Fig. 2A) and loss (G″) (Fig. 2B) moduli over a strain range of 1 and 10 %, indicating the LVE region to be about 10 %. As seen in Fig. 2A and B, all the hummus samples exhibited both G′ and G″ confirming their viscoelastic nature, however, elevated G′ values which were significantly higher than G″ were observed in all experimental hummus samples. This indicates that the experimental HPP-treated hummus samples exhibited gel behavior at the LVR contributing to their viscoelastic properties and the insignificant influence of G'' [2]. The observed G' > G″ phenomenon agrees with previous studies of commercial Spanish and HPP-treated hummus [2]. Additionally, the values for both storage and loss moduli were dependent on the HPP hold time with 5 min, 4 min, and 3 min being the highest in descending order which agrees with previous studies of HPP-treated hummus samples [16,48]. However, no significant difference was seen in the linearity trends of 0 min, 1 min, and 2 min samples as indicated by the values of G′ and G''.

**Fig. 3:**
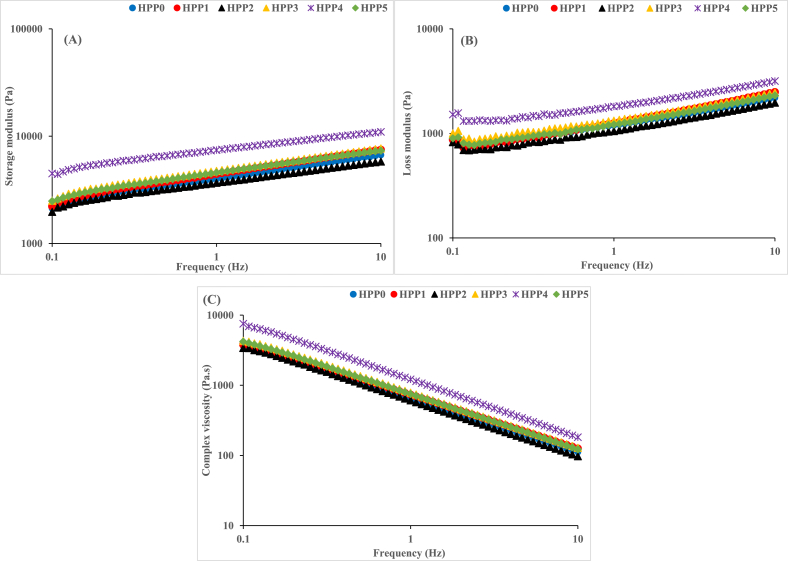
Viscoelastic properties of hummus samples treated with 350 MPa HPP. **HPP 0:** no treatment (control), **HPP 1:** treatment for 1 min, **HPP 2:** treatment for 2 min, **HPP 3:** treatment for 3 min, **HPP 4:** treatment for 4 min, **HPP 5:** treatment for 5 min.

The quantitative evaluation of the viscoelastic properties including storage (G′), loss (G″), and complex moduli of the HPP-treated hummus as a function of frequency (0.1–10 Hz) are presented in Fig. 3A–C. The frequency sweep test gives comprehensive information on the internal forces facilitating cross-linking in materials and their interaction with other components [49]. As can be seen from Fig. 3, G′ (Fig. 3A) and G″ (Fig. 3B) moduli consistently increased with an increase in applied frequency, yet G′ had prominently higher values than G″ in all the experimental hummus samples throughout the examined frequency range, which agrees with the results of the strain sweep test. This can be attributed to the effect of increased cross-linking density within the molecules of hummus caused by the HPP treatment, which caused protein aggregation, starch gelatinization, and coagulation [50]. Also, this is indicative of the solid-like properties of hummus samples contributing more to their elastic properties than viscous properties. The present results agree with a previous study [16]which documented an elevated G′ in hummus samples treated with 600 MPa. Similar findings were also observed in sterilized and unsterilized hummus paste [49]. Comparable results were reported in commercial Spanish hummus samples, although a plateau described at a lower frequency by the authors was not observed in the present study [2]. It is probable that the absence of this plateau could have been due to variations in hummus composition and processing. It was notable that the 4 min samples showed significantly higher G′ and G″ values compared to other samples, which may have resulted from use of the optimal HPP sample hold time. Additionally, there was no significant difference in the frequency-dependent behaviour of 1 min, 3 min, and 5 min samples as indicated by their G′ and G″ values. Furthermore, the 2 min samples showed consistently lower values of G′ and G″ which was comparable to the control throughout the frequency range. These variations in the viscoelastic behaviour can be attributed to the properties of the gel network formed due to the HPP-denatured chickpea protein components and sesame seed oil used during hummus preparation [51].

In contrast with storage and loss moduli, the complex modulus measures the ratio of the applied strain to measured stress, establishing the resistance of the sample to deformation. Unlike the storage and loss moduli behaviour, the complex modulus (Fig. 3C) decreased with an increase in the applied frequency. Specifically, all samples depreciated within the range of 10^4^ and 10^2^ with the 4 min-treated hummus sample exhibiting the least depreciation while the 2 min sample had the highest. The variations in the behaviour of the samples can be attributed to the density and strength of the gel network formed within the hummus sample because of the interactions between denatured proteins of the chickpea and sesame seed oil with other hummus components. Similar findings have been reported in previous studies [2,47].

**Fig. 4:**
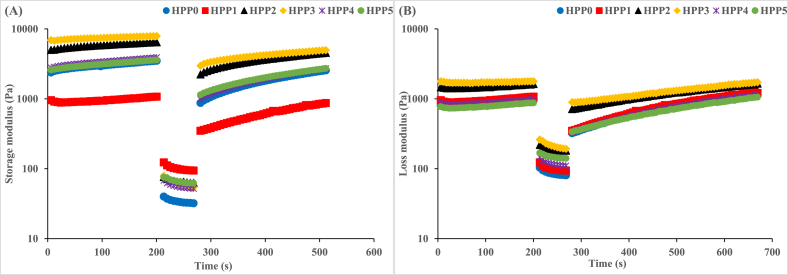
Thixotropic behaviors of hummus samples treated with 350 MPa HPP. **HPP 0:** no treatment (control), **HPP 1:** treatment for 1 min, **HPP 2:** treatment for 2 min, **HPP 3:** treatment for 3 min, **HPP 4:** treatment for 4 min, **HPP 5:** treatment for 5 min.

Thixotropy is a fundamental rheological phenomenon that measures the flow behaviour of materials and their response to deformation [52]. The thixotropic properties of the HPP-treated hummus samples were evaluated with a 3-step oscillation test corresponding to the response to very low shear stress, structural breakdown due to applied high shear stress, and structural recovery under a repeated very low shear stress, and the results are presented in Fig. 4 in the form of storage (G′) and loss (G″) moduli. Overall, all the evaluated hummus samples showed distinct time-dependent behaviour evinced by the structural regeneration to almost their initial G′ and G″ values following the removal of the applied high shear stress. It is worth noting that G′ (Fig. 4A) was consistently dominant over G″ (Fig. 4B) throughout the time under observation. Similar findings have been reported in commercial Spanish hummus [2], HPP-treated hummus packed in polylactide/essential oil blend film [53], HPP-treated defatted chickpea flour suspension [52], and HPP-treated chickpea flour slurry and paste [54].

### 3.7 Effect of HPP on hummus sensory characteristics

The sensory analysis (Table 5) indicated no significant difference (*p* > 0.05) in colour, taste, texture, flavour and overall acceptability between the control and samples treated with 350 MPa from 1 to 5 min. The mean scores for appearance, odour, taste, and overall acceptability were similar to those obtained in a previous study conducted on hummus to which potassium sorbate/vinegar/garlic/natamycin were added [55].

**Table 5:** Sensory evaluation of control and 350 MPa HPP hummus samples treated from 1 to 5 min.

Characteristic	Time (min)						
	0	1	2	3	4	5	*p* - value
Color	7.2 ± 0.2	7.4 ± 0.2	7.4 ± 0.3	7.4 ± 0.2	7.2 ± 0.2	7.0 ± 0.2	0.610 (NS∗)
Taste	6.8 ± 0.2	7.0 ± 0.2	6.8 ± 0.2	7.3 ± 0.2	7.0 ± 0.2	6.6 ± 0.3	0.290 (NS)
Texture	6.7 ± 0.2	6.5 ± 0.3	6.7 ± 0.3	6.7 ± 0.2	6.6 ± 0.3	6.4 ± 0.2	0.970 (NS)
Flavor	6.8 ± 0.2	6.9 ± 0.3	6.6 ± 0.3	7.1 ± 0.2	6.9 ± 0.2	6.4 ± 0.2	0.375 (NS)
Overall acceptability	6.9 ± 0.2	7.0 ± 0.2	6.9 ± 0.2	7.2 ± 0.2	6.9 ± 0.3	6.5 ± 0.2	0.295 (NS)

∗ Not significantly different. Differences were assessed at *p* < 0.05 among the means.

## 4 Conclusion

HPP at 350 MPa for 5 min was an effective treatment for the destruction of pathogens and to control hummus microbial levels. However, after HPP treatment, storage at 4^o^C is recommended. HPP treatment at 350 MPa for 2–5 min followed by storage at 4^o^C can maintain the shelf life of the product up to 28 d. This HPP treatment did not compromise on the texture, rheological properties, lipid oxidation or colour. Although requiring an initial heavy investment in machinery, this method would prove cost-effective for producing hummus in large batches over the long term. It is a good preservation technique for hummus producers to adopt.

## CRediT authorship contribution statement

**Tareq M. Osaili:** Writing – review & editing, Writing – original draft, Supervision, Resources, Project administration, Methodology, Investigation, Conceptualization. **Dinesh Kumar Dhanasekaran:** Writing – review & editing, Project administration, Methodology, Investigation, Formal analysis. **Fayeza Hasan:** Writing – review & editing, Writing – original draft, Visualization, Validation. **Reyad S. Obaid:** Writing – review & editing, Software, Resources, Project administration. **Anas A. Al-Nabulsi:** Writing – review & editing, Project administration, Investigation. **Amin N. Olaimat:** Software. **Leila Cheikh Ismail:** Validation, Supervision. **Hayder Hasan:** Writing – original draft. **Mutamed Ayyash:** Writing – original draft, Funding acquisition. **Gafar Babatunde Bamigbade:** Methodology, Investigation. **John Ortiz:** Project administration, Methodology. **Richard Holley:** Writing – review & editing.

## Declarations

This study was reviewed and approved by the University of Sharjah ethics committee with the approval number: 23-08-28-02-F, dated September 13, 2023. All participants provided written informed consent to participate in the study and for their data to be published. The authors have nothing to declare/competing interests.

## Data availability

Data will be made available on request. For requesting data, please write to the corresponding author.

## Declaration of competing interest

The authors declare that they have no known competing financial interests or personal relationships that could have appeared to influence the work reported in this paper.
