# Supplementary material for: High pressure processing of hummus: Enhancing microbial safety and stability, and reducing lipid oxidation
Source: Heliyon. 2025 Feb 10;11(4):e42590. doi: 10.1016/j.heliyon.2025.e42590 (PMC11876884; doi:10.1016/j.heliyon.2025.e42590)
Supplement: Multimedia component 1 [file mmc1.pdf]

Title of the study involving human participant(s): Enhancing Hummus Safety Through High Pressure Processing: Microbial, Physical, and Sensory Evaluation

Author Name(s): Tareq M. Osaili1,2,3, Dinesh Kumar Dhanasekaran2, Fayeza Hasan2, Reyad S. Obaid1,2, Anas A. Al-Nabulsi3, Amin N. Olaimat4, Leila Cheikh Ismail1,2, Hayder Hasan1,2, Mutamed Ayyash5, Gafar Babatunde Bamigbade5, John Ortiz6, Richard Holley7

Institution(s): 1Department of Clinical Nutrition and Dietetics, College of Health Sciences, University of Sharjah, P. O. Box 27272 Sharjah, United Arab Emirates
2Research Institute of Medical & Health Sciences, University of Sharjah, P. O. Box 27272 Sharjah, United Arab Emirates
3Department of Nutrition and Food Technology, Faculty of Agriculture, Jordan University of Science and Technology, P.O. Box 3030, Irbid 22110, Jordan.
4Department of Clinical Nutrition and Dietetics, Faculty of Applied Medical Sciences, The Hashemite University, Zarqa, Jordan
5Department of Food, Nutrition and Health, College of Food and Agriculture, United Arab Emirates University (UAEU), United Arab Emirates.
6Smartfood Solutions FZCO, Dubai Silicon Oasis, Dubai, United Arab Emirates, P.O. Box 34114[illegible]
7Department of Food Science and Human Nutrition, University of Manitoba, Winnipeg, Manitoba R3T 2N2, Canada

Date:
06/02/2024

I, the undersigned author of the above-mentioned study, hereby declare the following:

1. I have obtained written informed consent from the participant(s) / patient(s) for the publication of this study, any accompanying data and images. Where consent was obtained from someone other than the participant(s) / patient(s), I confirm that this proxy was authorised to provide consent on the participant's / patient's behalf.
2. Where the participant(s) / patient(s) is/are a minor(s), we followed local laws on the age and circumstances under which they may consent for themselves. If they were not of legal age to consent, consent was obtained from an authorised proxy i.e., the parents or legal guardian(s). If the minor(s) has/have reasonable understanding of the informed consent and implications, signature (or assent, as appropriate) was also obtained from the minor(s).
3. Where the participant(s) / patient(s) provided consent themselves, I confirm that they had capacity to do so, and any mental or physical disabilities were taken into consideration in the process of informing and obtaining written informed consent.
4. Where the participant(s) / patient(s) has/have died, I confirm that the consent given still allows for publication.
5. I confirm that all content presented in this study, associated data and images have been deidentified and anonymized to the best possible extent.
6. The original signed and dated consent form is held by the treating institution or appropriate governing local / regional / national body and will be retained according to the policies and procedures of the institution or governing body.
7. The written informed consent form (please **<u>do not</u>** include with your submission) includes all relevant information pertinent to each participant / patient (such as the name, age, condition, medical history, diagnosis, and treatment)
8. The participant(s) / patient(s) / authorized proxy were fully informed of the purpose of this study, the potential risks and benefits of publication, and the consequence of disclosing their personal information.
9. The participant(s) / patient(s) or authorized proxy were given the opportunity to ask questions regarding publication of the study, had their questions answered fully and have consented to publish all associated data and images. In the case of clinical studies, the participant(s) / patient(s) or authorized proxy approved the final version of the manuscript.
10. The participant(s) / patient(s) or legal guardian(s) were informed that their consent and participation in the publication of this study is entirely voluntary and that they have the right to withdraw their consent at any time.
11. If this is a clinical study manuscript, I confirm that at least one of the authors of this paper was involved in the care of the participant(s) / patient(s).
12. I confirm that my article complies with the appropriate local / regional / national law on consent and privacy.

By signing this declaration form, I acknowledge that I have read and understood the information provided above, and I attest to the accuracy of this declaration. I understand that any false or misleading information may result in the rejection of the manuscript or other disciplinary actions.

As corresponding author, I hereby declare that I sign this document on behalf of all the authors of the above-mentioned study involving human participants.

Corresponding author's signature:	tareq osaili
Date:	06/02/2024

Please submit this **Human Participant Declaration Form** along with the manuscript to the journal. **<u>Note:</u>** The written informed consent form must NOT be submitted with your manuscript but must be made available to the journal if specifically requested.

Please retain a copy of this declaration for your records.
