# Supplementary material for: High pressure processing of hummus: Enhancing microbial safety and stability, and reducing lipid oxidation
Source: Heliyon. 2025 Feb 10;11(4):e42590. doi: 10.1016/j.heliyon.2025.e42590 (PMC11876884; doi:10.1016/j.heliyon.2025.e42590)
Supplement: Multimedia component 2 [file mmc2.pdf]

Scorecard-Hedonic rating scale

Sample code:

Name:

Panelist no:

Taste the given coded sample and tick (X) how much you like or dislike it on the point in the scale which best describes your feeling.

Score	Color	Taste	Texture	Flavor	Overall acceptability
(9) Like extremely					
(8) Like very much					
(7) Like moderately					
(6) Like lightly					
(5) Neither like or dislike					
(4) Dislike lightly					
(3) Dislike moderately					
(2) Dislike very much					
(1) Dislike extremely					
